# Comparison between headless cannulated screws and partially threaded screws in femoral neck fracture treatment: a retrospective cohort study

**DOI:** 10.1038/s41598-021-03494-3

**Published:** 2022-02-02

**Authors:** Yilin Wang, Na Han, Dianying Zhang, Peixun Zhang, Baoguo Jiang

**Affiliations:** 1 Department of Orthopedics and Traumatology, Peking University People’s Hospital, Beijing, People’s Republic of China; 2 The Key Laboratory of Trauma and Neural Regeneration (Peking University), Ministry of Education, Beijing, People’s Republic of China; 3 National Center for Trauma Medicine, Beijing, People’s Republic of China

**Keywords:** Trauma, Risk factors, Outcomes research

## Abstract

The choices of the treatments for femoral neck fractures (FNF) remain controversial. The purpose of this study is to evaluate the prognoses of the variable pitch fully threaded headless cannulated screws (HCS) in the fixation of femoral neck fractures and to compare them with those of partially threaded cannulated screws (PCS). Between 1st January 2012 and 31st December 2016, there were 89 patients with the main diagnose of FNF who accepted the treatment of closed reduction cannulated screw fixation in Peking University People’s Hospital. 34 cases of PCS and 23 cases of HCS met the criterion. The characteristics, prognoses and the imaging changes of all cases were described and the differences between the two groups were compared. Statistical analyses were performed using SPSS version 23.0 (SPSS Inc., USA). Mann–Whitney U test, Analysis of Variance and Chi-square test were used. Statistical significance was defined as *P* value (two sided) less than 0.05. There was no significant difference in the general characteristics, fracture classifications and reduction quality between the two groups. HCS group had a significant lower angle decrease rate (30.4% vs. 58.8%, *P* = 0.035), femoral neck shortening rate (26.1% vs. 52.9%, *P* = 0.044) and screw back-sliding rate (21.7% vs. 50.0%, *P* = 0.032), but a higher screw cut-out rate (21.7% vs. 0.0%, *P* = 0.008). In non-displacement fracture subgroup, HCS had significant higher Harris Score (92 vs. 90, *P* = 0.048). Compared with PCS, HCS had a lower screw back-sliding rate, femoral shortening rate, angle decrease rate and similar function score, but would result in more screw cut-outs in displaced FNF. As a conclusion, HCS should not be used in displaced FNF due to its higher screw cut-out rate, and its potential advantage in non-displaced FNF needs to be further proved. Further qualified investigations with a larger scale of patients and longer follow-up are needed in the future.

## Introduction

Femoral neck fractures (FNF) are very common and often result in significant morbidity and mortality, hence it is important to identify the type and characteristics of the fracture as early as possible, and to choose the appropriate treatment according to the patient's specific conditions. The present treatments for FNF are controversial. A variety of risk factors affect the prognosis^1^. Therefore, it is important to study the treatment methods of FNF.

The main treatment methods for FNF are hip arthroplasty, fracture reduction internal fixation and conservative treatment. A large number of studies have shown that three partially threaded cannulated screws placed in parallel with inverted triangles have good biomechanical performance and good clinical efficacy^2–6^. For elderly patients with multiple comorbidities, internal fixation is painless, convenient and minimal invasive compared with hip replacement. It is also more economical and easier to operate than other fixation methods, thus internal fixation could be the first choice for FNF in many circumstances. However, some patients experienced postoperative screws back-sliding, hip varus and femoral neck shortening, which affected the treatment effect^7–12^. Although the shortening of the femoral neck was not considered as a failure of internal fixation, it decreased the patients’ mobility and quality of life intensively^7–9,13,14^. In addition, the complications associated with cannulated screws such as nonunion and avascular necrosis still remained unsolved. Further discussion is needed on the indications for internal fixation, the risk factors of complications, and the precautions against the complications^15^.

The variable pitch fully threaded headless cannulated screw (HCS) (Acutrak 6/7, ACUMED) was born in the 1990s. It is a headless, conical, fully threaded, variable pitch screw. From the head to the tail, the pitch changes from large to small, which results in a faster entering speed of the head than the tail, thereby compressing the fracture as the screw enters the bone. The headless design allows the screws to be implanted into the bone surface, which may reduce the irritation to soft tissues^16^. A lot of biomechanical studies have been carried out around the screw^6,17,18^, and have confirmed its better biomechanical performance than other compression screws in vitro^19–21^. Clinically, HCS is often used for in situ fixation of some fractures, such as scaphoid fractures and ankle fractures^22,23^. In recent years, the application of HCS has been promoted into the fixation of fractures in various parts of the body and some joint fusion surgeries^24–27^. This screw is both minimally invasive and biomechanically strong, and therefore offers a new possible solution to the femoral neck shortening and hip varus after FNF. However, the current clinical research on this screw is relatively scarce, and its fixation effect remains controversial^28,29^.

HCS was first used to treat FNF in 2012 in the traumatic orthopedics department of Peking University People’s Hospital. Between 1st January 2012 and 31st December 2016, there were 89 patients with the main diagnose of FNF who accepted the treatment of closed reduction cannulated screws fixation in the hospital. Among them 53 patients used the partially threaded cannulated screws (PCS) (7.3 mm, AO foundation), and 36 used HCS. This study intends to describe the prognosis of patients with FNF after cannulated screw internal fixation, to compare HCS with PCS, and to help to evaluate the effect of HCS in FNF.

## Materials and methods

This study was a retrospective cohort study. All patients' medical records, imaging data and postoperative follow-up information were collected. The study was conducted in accordance with the Declaration of Helsinki. The protocol was approved by the Ethics Committee of Peking University People’s Hospital (Project identification code 2019PHB160). The requirement for informed consent was waived by the Ethics Committee of Peking University People’s Hospital because of the retrospective cohort nature of the study.

### Inclusion criteria

Patients admitted between 1st January 2012 and 31st December 2016, with the main diagnose of FNF, accepted closed reduction cannulated screws fixation, with the implant of three PCS or HCS (7.3 mm, Acutrak 6/7, ACUMED).

### Exclusion criteria

Open fracture, open reduction, multiple injuries, pathological fracture, severe complications of other systems or death during the follow-up period, second trauma or fracture of the operated place, loss to follow-up or follow-up time less than 1 year.

### Surgery and follow up

All operations aimed to fix the fracture by closed reduction minimally invasively. The operations were performed on a traction table under the help of a C-arm machine. The patient was supine with traction along the long axis of the injured femur. Following traction, the leg was internally rotated to achieve a good reduction. Three parallel guidewire were placed using an aiming device in a triangle position, and then three small incisions were made to allow the screws drilling into the femoral neck.

The patients took the hip antero-posterior (AP) and lateral (LAT) X-rays within 5 days after the operations to record the initial position of the screws and the reduction of the fracture, and the first radiology measurements were performed. All patients were required to take postoperative AP and LAT hip X-rays at 6 weeks, 12 weeks, 6 months and 1 year after the operation. Once the sign of union (the blurring of the fracture line) appeared in 6–8 weeks, the patients would be required to start to bear partial weight. Full weight bearing was prohibited within 12 weeks. A patient could be diagnosed as fracture union by painless full weight bearing walking or by radiology. If the fracture was not healed within 1 year, it would be considered to be fracture nonunion^30,31^. At the 1-year follow-up, the AP and LAT hip X-rays were taken and measured again. Patients with hip pain were recommended to examine hip MR and hip CT to determine whether there was screw cut-out or femoral head necrosis. The Harris scores were also measured at 1-year follow-up^32^.

### Imaging measurements

All imaging data were measured by three qualified orthopedic doctors. The continuous variables were averaged, and the value of categorical variables was decided by the majority.

The Garden classification, Pauwels classification and cortical thickness index (CTI)^33,34^ were evaluated on the preoperative anterior–posterior hip X-ray.

All postoperative measurements were performed in the General Electric company's Centricity Picture Archiving and Communication Systems (PACS), which could measure the accurate angle and imaging distances. All imaging length data were standardized by comparing to the imaging length of the screw whose actual length was known to correct the leg rotation and magnification. First the imaging length of the screw was measured, and each coefficient of each X-ray film was calculated by dividing the actual length by the imaging length. The standardized length was obtained by using the imaging length to multiply the aforementioned coefficient. All length data appeared in the study have been standardized.

**Figure 1:**
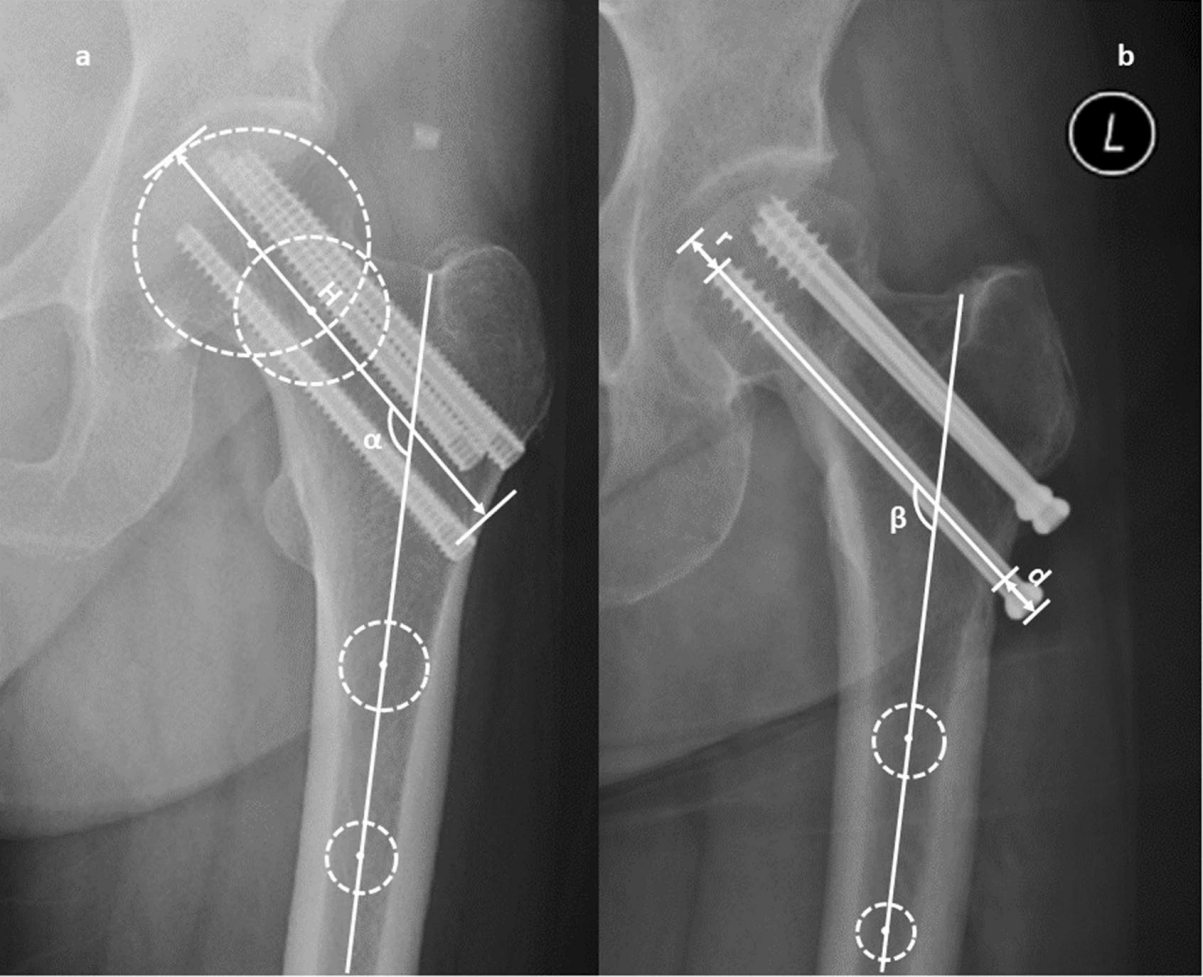
Imaging measurement after FNF cannulated screw fixation. (**a**) The measurement of the length of the femoral neck axis and the neck-shaft angle. H was the length of the femoral neck axis, and α was the neck-shaft angle. (**b**) The distance from the screw head to the femoral head cortex and the distance between the screw tail and the lateral cortex. The average screw migration distance and the average screw back-sliding distance can be obtained by calculating the difference. (The figure was created using Microsoft Office 2019, see aka.ms/msoffices).

As shown in Fig. 1a, the axis of the femoral shaft was determined by connecting the center of two circles tangent to the medial cortex of the femoral shaft. The center of the femoral head was determined by the center of the best-fit circle. The axis of the femoral neck was determined by connecting the center of the femoral head to the center of the circle tangent to the cortex of femoral neck^35,36^. H was the length of the femoral neck axis^36^, the initial measurement value was H, the postoperative 1 year measurement value was H′. Delta H = H–H′ was the change of femoral neck length. The angle between the two axes indicated by α in Fig. 1a was the neck-shaft angle. The initial measurement value was α, and the angle value measured 1 year after surgery was α'. Delta α = α–α' was the change of the neck-shaft angle.

As shown in Fig. 1b, r was the distance from the screw head to the femoral head cortex (along the axis of the screw). The distances of the three screws from the inside to the outside were recorded as r1, r2, r3. The average value of the initial measurement was recorded as Ra, and the measurement 1 year later was recorded as Ra’. The average screw migration distance was Delta Ra, Delta Ra = Ra–Ra′. D was the distance between the screw tail and the lateral cortex. The values of the three screws from the inside to the outside were d1, d2 and d3, and the average value was also calculated. The average value of the initial measurement was recorded as Da, and the measurement after 1 year was Da′. The average screw back-sliding distance was Delta Da, Delta Da = Da–Da’. The angle between the two axes indicated by β in Fig. 1b was the trajectory angle. The mean value of three screws in the first postoperation X-ray was used in the analysis.

The Garden alignment index was used to assess the levels of reduction, which was evaluated from the first hip X-ray after surgery^37,38^. The degree within the range of 155–180 degree in both anteroposterior and lateral views was considered acceptable, otherwise was unacceptable.

All the screws were distributed in triangle position. The position was identified as non-inverted triangle if there was a gap between the upper two screws in the AP x-ray. On the contrary, the positions in Fig. 1a, b were identified as inverted triangle.

### Statistics analysis

The influencing factors included: gender, age, body mass index (BMI), type of internal fixation, length of hospital stays, time from injury to surgery, CTI, Pauwels classification, Garden classification and Garden index. The prognostic indicators included: femoral head necrosis rate, screw cut-out rate, nonunion rate, Harris score, the good and excellent rate of Harris score, femoral neck shortening, the average screw migration distance, the average screw back-sliding distance, the change of neck-shaft angle, femoral neck shortening rate, femoral neck-shaft angle decrease rate, screw back-sliding rate. The femoral neck shortening no less than 5 mm was classified as femoral neck shortening to calculate the rate. The decrease of neck-shaft angle no less than 5° was classified as neck-shaft angle decrease to calculate the rate. The average back-sliding distance no less than 3 mm was classified as the screw back-sliding to calculate the rate.

The prognoses of all cases were described, meanwhile the differences of the two implant groups were compared. Statistical analyses were performed using SPSS version 23.0 (SPSS Inc., USA). After the test of normality, Mann–Whitney U test, analysis of variance and Chi-square test were used to determine the risk factors respectively. In the Chi-square test, the Pearson chi-square was used when the theoretical numbers T was no less than 5 and total sample size n was no less than 40, otherwise the Fisher’s exact test was used. Significant difference was considered when *P* value (two sided) was less than 0.05.

Femoral neck necrosis and screw cut-out were analyzed using a multivariate logistic regression model. Age, gender, BMI, CTI, time from injury to operation, length of hospital stay, type of internal fixation, Garden classification, Pauwels classification, Garden index level, screw position and trajectory angle were the independent variables included in the model. To prevent the issue of multiple collinearities in the multivariate analysis, a Spearman correlation analysis was performed among the various factors. Furthermore, in order to identify the most important influencing factors, the Backward Wald method was used.

### Ethics approval

The study was conducted in accordance with the Declaration of Helsinki. The protocol was approved by the Ethics Committee of Peking University People’s Hospital (Project identification code 2019PHB160). The requirement for informed consent was waived by the Ethics Committee of Peking University People’s Hospital because of the retrospective cohort nature of the study.

## Results

A total of 89 patients met the inclusion criteria. We excluded 4 patients with multiple trauma, 1 patient with postoperative pulmonary embolism, 1 patient with liver cancer, 1 patient with severe heart disease, 1 patient with postoperative trauma, 10 patients with the follow-up time less than 1 year and 14 patients without follow-up. Finally, 34 cases of PCS (referred to as PCS group) and 23 cases of HCS (referred to as HCS group) were included. There was no missing data.

### General characteristics

There were 24 male patients, accounting for 42.1% of the all patients, 16 male patients in the PCS group and 8 male patients in the HCS group. The mean age of the patients was 59.8 ± 15.0 (66.2) years. The average BMI of the patients was 23.2 ± 3.4 (19.3). Notice here the number in bracket represents the range of the variable. There was no significant difference of the percentage of male (*P* = 0.357), the mean age (F1, 55 = 0.036, *P* = 0.85), the mean BMI (F1, 55 = 3.303, *P* = 0.075), and the median CTI (Z =  − 0.992, *P* = 0.321) between two groups. The median length of hospitalization was 9.0 days. The median time from injury to surgery was 4.0 days. The median follow-up time was 640.5 days. There was no statistical difference of length of hospitalization (Z =  − 1.042, *P* = 0.297); the time from injury to surgery (Z =  − 1.424, *P* = 0.155); and the follow up time (Z =  − 0.903, *P* = 0.367) between the two groups. Overall comparison of the general characteristics of the two groups of patients, no significant difference was found (see Table 1).

**Table 1:** General characteristics of patients in different internal fixation groups.

Characteristics	PCS group (n = 34 (59.6%))	HCS GROUP (n = 23 (40.4%))	*P* value
Gender (Male) (n (%))	16 (47.1%)	8 (34.8%)	0.357
Age (years, mean ± SD (range))	60.2 ± 15.5 (66.2)	59.4 ± 14.5 (50.7)	0.850
BMI (mean ± SD (range))	22.5 ± 2.7 (12.0)	24.1 ± 4.1 (18.5)	0.075
CTI (median (IQR))	0.55 (0.10)	0.53 (0.06)	0.321
Length of Hospital Stay (days, median (IQR))	8.0 (4.5)	10 (4.0)	0.297
Time from injury to operation (days, median (IQR))	3.5 (4.0)	4.0 (6.0)	0.155
Follow up time (days, median (IQR))	640.5 (442.25)	635.0 (308.0)	0.367

SD and IQR stand for standard deviation and interquartile range respectively.

### Fracture classifications and reduction quality

The distribution of Garden classification and Pauwels classification of all patients was shown in Table 2. There were 24 cases of Garden I type, accounting for 42.1%, including 15 cases of PCS group, 9 cases of HCS group; 10 cases of Garden II type, accounting for 17.5%, including 5 cases of PCS group and 5 cases of HCS group; 17 cases of Garden III type, accounting for 29.8%, including 11 cases of PCS group, 6 cases of HCS group; 6 cases of Garden IV type, accounting for 10.5%, including 3 cases of PCS group and 3 cases of HCS group; There were 11 cases of Pauwels type I, accounting for 19.3%, including 10 cases of PCS group and 1 case of HCS group; 32 cases of Pauwels type II, accounting for 56.1%, including 18 cases of PCS group and 14 cases of HCS group; 14 cases of Pauwels type III, accounting for 24.6%, including 6 cases of PCS group and 8 cases of HCS group. In order to increase the statistical efficiency, Garden I-II, Garden III-IV and Pauwels I-II were combined separately, and the chi-square test was used to compare the difference between the two groups. There was no significant difference of Garden classification (*P* = 0.877) and Pauwels classification (*P* = 0.140).

**Table 2:** Fracture classification of patients in different internal fixation groups.

Fracture classifications	PCS group (n = 34 (59.6%))	HCS group (n = 23 (40.4%))	*P* value
**Garden classification**			
I–II (n (%))	20 (58.8%)	14 (60.9%)	0.877
III–IV (n (%))	14 (41.2%)	9 (39.1%)	
**Pauwels classification**			
I–II (n (%))	28 (82.4%)	15 (65.2%)	0.140
III (n (%))	6 (17.6%)	8 (34.8%)	

As shown in Table 3, there was no significant difference of the Garden Index level (*P* = 1.000), AP Garden Index (Z =  − 0.237, *P* = 0.813) and LAT Garden Index (Z =  − 0.017, *P* = 0.987) between the two groups.

**Table 3:** Reduction quality in different internal fixation groups.

Reduction quality	PCS group (n = 34 (59.6%))	HCS group (n = 23 (40.4%))	*P* value
**Garden index level**			
Acceptable (n (%))	29 (85.3%)	20 (87.0%)	1.000
Unacceptable (n (%))	5 (14.7%)	3 (13.0%)	
AP Garden index (median (IQR))	165.0 (9.25)	163.0 (9.0)	0.813
LAT Garden index (median (IQR))	178.0 (5.0)	178.0 (5.0)	0.987

IQR stands for interquartile range.

### Postoperative imaging data

As shown in Table 4, there was no significant difference in terms of the proportion of cases with the inverted triangle screws position (*P* = 0.413) and trajectory angle (F1, 55 = 2.235, *P* = 0.141) between two groups. There was significant difference of the change of neck-shaft angle (F1, 55 = 5.435, *P* = 0.023) and the average screw back-sliding distance (Z =  − 2.033, *P* = 0.042). Meanwhile, no significant difference was found in the average screw migration distance (Z =  − 0.293, *P* = 0.770) and femoral neck shortening (Z =  − 1.057, *P* = 0.290).

**Table 4:** Postoperative imaging changes in different internal fixation groups.

Imaging data	PCS group (n = 34 (59.6%))	HCS group (n = 23 (40.4%))	*P* value
Screw position as inverted triangle (n (%))	20 (58.8%)	11 (47.8%)	0.413
Trajectory angle (degree, mean ± SD (range))	139.3 ± 7.7 (40)	142.8 ± 9.5 (37)	0.141
Change of neck-shaft angle (degree, mean ± SD (range))	6.0 ± 6.0 (27)	2.6 ± 4.0 (16)	0.023
Average screw back-sliding distance (mm, median (IQR))	2.7 (5.6)	1.2 (4.1)	0.042
Average screw migration distance (mm, median(IQR))	1.5 (3.2)	1.1 (4.6)	0.770
Femoral neck shortening (mm, median (IQR))	5.1 (7.6)	3.2 (5.2)	0.290

SD and IQR stand for standard deviation and interquartile range respectively.

### Prognostic indicators

As shown in Table 5, there was significant difference in the cut-out rate (*P* = 0.008), angle decrease rate (*P* = 0.035), femoral neck shortening rate (*P* = 0.044), and screw back-sliding rate (*P* = 0.032). No significant difference was found in the nonunion rate (*P* = 0.159), femoral head necrosis rate (*P* = 0.744), Harris score (Z =  − 0.230, *P* = 0.818) and the good and excellent rate of Harris score (*P* = 0.443).

**Table 5:** Prognoses of patients with different internal fixation groups.

Prognoses	PCS group (n = 34 (59.6%))	HCS group (n = 23 (40.4%))	*P* value
Screw cut-out rate (n (%))	0 (0.0%)	5 (21.7%)	0.008
Angle decrease rate (n (%))	20 (58.8%)	7 (30.4%)	0.035
Femoral neck shortening rate (n (%))	18 (52.9%)	6 (26.1%)	0.044
Screw back-sliding rate (n (%))	17 (50.0%)	5 (21.7%)	0.032
Nonunion rate (n (%))	0 (0.0%)	2 (8.7%)	0.159
Femoral head necrosis rate (n (%))	8 (23.5%)	4 (17.4%)	0.744
Harris score (median (IQR))	90 (10)	90 (16)	0.818
Excellent and good rate of Harris score (n (%))	28 (82.4%)	17 (73.9%)	0.443

IQR stands for interquartile range.

### Multivariate logistic analysis and subgroup analysis

A Spearman correlation analysis was performed among the various factors, and none of the absolute value of the correlation coefficients was higher than 0.6. For femoral head necrosis, three significant variables were selected. The Garden classification type III-IV (fracture displacement), the Garden index level III-IV (unsatisfactory reduction), and smaller the trajectory angle were the three most important risk factors for femoral head necrosis. The overall accuracy of the model was 87.7% (see Table 6). For screw cut-outs, one meaningful variable was selected. The Pauwels classification type III was the most important risk factors for screw cut-outs. The overall accuracy of the model was 91.2% (see Table 7).

**Table 6:** The multivariate logistic regression analysis of the risk factors for femoral head necrosis.

Risk factors	Coefficient	Standard error	Wald test statistic	*P* value	Odds ratio (OR)	95% confidence interval for OR
Garden classification type III-IV (fracture displacement)	4.111	1.541	7.118	0.008	61.012	2.977–1250.426
Garden index level III-IV (unsatisfactory reduction)	2.266	1.110	4.170	0.041	9.641	1.095–84.848
Trajectory angle	-0.234	0.104	5.008	0.025	0.792	0.645–0.971

**Table 7:** The multivariate logistic regression analysis of the risk factors for screw cut-out.

Risk factors	Coefficient	Standard error	Wald test statistic	*P* value	Odds ratio (OR)	95% confidence interval for OR
Pauwels classification type III	2.821	1.172	5.794	0.016	16.8	1.689–167.109

Fracture displacement could have significant impact on prognosis, therefore we performed a subgroup analysis to compare the prognoses of two groups in non-displaced fractures (Garden I–II). Results show that HCS group had higher median Harris scores (Z =  − 1.981, *P* = 0.048), which could be a potential advantage compared to PCS in non-displaced fractures (see Table 8). In displaced fractures (Garden III–IV), HCS group had higher cut-out rate (*P* = 0.004) (see Table 9). This result suggested that HCS might not be recommended in displaced FNF.

**Table 8:** Prognoses of patients with different internal fixation groups in non-displaced fractures.

Prognoses	PCS group (n = 20 (58.8%))	HCS group (n = 14 (41.2%))	*P* value
Screw cut-out rate (n (%))	0 (0.0%)	0 (0.0%)	–
Angle decrease rate (n (%))	11 (55.0%)	6 (42.9%)	0.486
Femoral neck shortening rate (n (%))	8 (40.0%)	2 (14.3%)	0.141
Screw back-sliding rate (n (%))	7 (35.0%)	2 (14.3%)	0.250
Nonunion rate (n (%))	0 (0.0%)	0 (0.0%)	–
Femoral head necrosis rate (n (%))	2 (10.0%)	0 (0.0%)	0.501
Harris score (median (IQR))	90 (7.8)	92 (6.0)	0.048
The excellent and good rate of Harris score (n (%))	18 (90.0%)	14 (100.0%)	0.501

IQR stands for interquartile range.

**Table 9:** Prognoses of patients with different internal fixation groups in displaced fractures.

Prognoses	PCS group (n = 14 (60.9%))	HCS group (n = 9 (39.1%))	*P* value
Screw cut-out rate (n (%))	0 (0.0%)	5 (55.6%)	0.004
Angle decrease rate (n (%))	9 (64.3%)	1 (11.1%)	0.029
Femoral neck shortening rate (n (%))	10 (71.4%)	4 (44.4%)	0.383
Screw back-sliding rate (n (%))	10 (71.4%)	3 (33.3%)	0.102
Nonunion rate (n (%))	0 (0.0%)	2 (22.2%)	0.142
Femoral head necrosis rate (n (%))	6 (42.9%)	4 (44.4%)	1.000
Harris score (median (IQR))	91 (20.0)	79 (22.0)	0.136
The excellent and good rate of Harris score (n (%))	10 (71.4%)	3 (33.3%)	0.102

IQR stands for interquartile range.

We also attempted to conduct subgroup analysis in Pauwels I–II fractures, but found no significant difference between two fixation groups. Therefore, the results are not reported here.

## Discussion

This study aimed to find a better solution for FNF fixation. In clinical practice, we noticed the better fixation strength of HCS, and hypothesized that HCS could decrease the femoral neck shortening rate and neck-shaft angle decrease rate. In a short summary, we first compared the general characteristics between two fixation groups and found no significant difference, confirming no selection bias. By comparing the imaging data, we found that HCS group did have a lower screw back-sliding rate, femoral shortening rate and angle decrease rate, and this met our hypothesis. However, HCS group had no advantage in the overall prognosis, but a higher screw cut-out rate. What resulted in a better imaging performance but a worse prognosis? We considered there to be one or more confounding biases. Multivariate logistic regression revealed that fracture displacement was a major risk factor on the necrosis. To eliminate the effects of fracture displacement, we conducted a subgroup analysis. Results showed no screw cut-out nor non-union cases in both fixation groups. Furthermore, HCS group had significant higher Harris score, which probably demonstrated a better curative effect in HCS group. By decreasing femoral neck shortening rate and angle decrease rate, HCS might preserve more hip joint function. On the contrary, in displaced FNF patients, HCS had more cut-out cases. The displaced FNF had more femoral neck shortening than non-displaced FNF. Since PCS allowed sliding while HCS did not, screw cut-out did not occur much although sliding occurred a lot in PCS group.

With the same purpose, Dr. Zlowodzki et al. raised the concern about femoral neck shortening after fracture fixation^7^. They found that femoral neck shortening after FNF fixation with multiple cancellous screws was common and it had a significant negative impact on physical functioning^8,9^. Under the motivation of finding solutions to prevent femoral neck shortening, researchers made their efforts to study different kinds of length stable implants including fully threaded cannulated screws^18,39–42^. The published papers on the use of fully threaded cannulated screws in the treatment of FNF were few with a relatively low evidence level. The clinical reports of HCS were even fewer (see Table 10). The studies were listed in order of evidence level from high to low, and the biomechanics studies were also included.

**Table 10:** Studies on fully threaded cannulated screws in the fixation of FNF.

Author	Study style	Internal fixation	Control group	Published year	Number of patients/specimens	Age (year)	Follow-up (month)	Conclusion
Guvenir Okcu^29^	Prospective randomized	Acutrak 6/7	6.5 or 7.3 mm partially threaded screws	2015	44	21–70	12–18	Partial-threaded cannulated screws offer a shorter union time and less complication rate
Baokun Zhang^43^	Biomechanics and prospective	Two Headless Cannulated Compression Screws plus an Ordinary Cannulated Screw	Ordinary cannulated compression screw	2018	20 models and 59 patients	20–65	10.7 ± 3.2	One OCCS plus two HCCSs in the treatment of vertical FNF produced better outcome than using OCCS alone
Chiang, M. H.^44^	Retrospective	Acutrak 6/7	7.3-mm partially threaded cannulated screws	2019	50	37–95	12.6–40.3	The FTHCSs may be a substitute for PTCSs, but it cannot prevent femoral neck shortening and varus collapse after fracture fixation
Yoram A. Weil^41^	Retrospective	7.3 mm titanium screws (Depuy Synthes, Solothurn, Switzerland)	6.5 mm titanium screws with a 22-mm thread length (Biomet Warsaw, IN, USA)	2018	65	14–91	12+	The addition of 2–3 fully threaded screws placed in parallel, inverted triangle configuration for FNFs can significantly decrease the amount of femoral neck shortening associated with the traditional fixation methods of these fractures using partially threaded screws
Lazaro, L. E.^45^	Prospective	Two fully threaded cannulated screws augmented with an endosteal fibular allograft	–	2016	27	29–84	17.4 ± 6.6	The fibular allograft reconstructs the comminuted femoral neck, and the osteointegration overtime increases the strength of the host bone–graft interface. This added strength seems to provide the stability needed to better preserve the intraoperative reduction, obtain good outcomes, and reduce the complications associated with FNF
Sreevathsa Boraiah^46^	Retrospective	Fully threaded screws coupled with either a DHS or DHHS	–	2010	54	48–100	15–36	Reduction with a stable calcar pivot, intraoperative compression and length-stable fixation can achieve high union rates with minimal femoral neck shortening and improved functional outcomes
Sreevathsa Boraiah^35^	Retrospective	Fully threaded screws coupled with either a DHS or DHHS	–	2010	54	48–100	9–30	Using intraoperative compression and length stable fixation, minimal shortening of the femoral neck with high union rates were achieved
Baokun Zhang^42^	Biomechanics	headless cannulated compression screw (Acumed)	Ordinary cannulated compression screw (Stryker)	2018	30	–	–	HCCS performs with better biomechanical stability than OCCS in the treatment of vertical FNF, especially with the Pauwels angle of 70∘
Jiantao Li^40^	Biomechanics on simulate 3D models	3-D models of PTS (6.5 mm diameter and 16 mm thread length) and FTS (6.5 mm diameter and fully thread length)	3-D models of PTS (6.5 mm diameter and 16 mm thread length) and FTS (6.5 mm diameter and fully thread length)	2018	–	–	–	For unstable FNF, superior results were obtained by stabilizing the fracture with triangular configuration formed by one superior PTS and two inferior FTSs when compared with other configurations of two FTSs and one PTS
Thomas K. Schaefer^18^	Biomechanics	7.3 mm cannulated screws, two partially threaded and one fully threaded (Synthes, Oberdorf, Switzerland)	Three partially threaded cannulated screws	2015	16	–	–	The construct with a fully threaded screw in the area of the posterior neck comminution showed significantly higher bending stiffness and less failure compared to the conventional partially threaded screws
Tim Alves^39^	Biomechanics	Three parallel fully threaded 6.5-mm screws	Three partially threaded 6.5-mm screws(parallel and nonparallel)	2010	21	–	–	HA bone substitute augmentation of fixation with 3 parallel partially threaded screws, and possibly 3 fully threaded screws alone, may be strong enough to resist femoral neck shortening following fracture fixation

The prospective randomized study published by Guvenir Okcu et al. in 2015 concluded that PCS could offer a shorter union time and a lower complication rate compared to HCS, while the functional scores were similar between groups^29^. Their overall results were similar to our study. We also found no significant difference between two groups in Harris score, but more nonunion and screw cut-out cases occurred in the HCS group. However, their study lacked further analysis on the reason of which factors influenced the prognosis and the role HCS plays under different conditions.

With a higher complication rate, HCS group patients should have worse functional scores. What were the reasons that HCS group had similar functional score despite higher complication rate? We proposed four possible reasons as follows.

First, certain factors that could be crucial to the prognoses including garden classification, reduction quality and bone mineral density were not considered in Dr. Okcu et al.’s study. In our study, femoral neck necrosis and screw cut-out were analyzed using a multivariate logistic regression model. Recognizing fracture displacement to be a major influence factor to the prognosis, we proceeded subgroup analysis in non-displaced fractures patients. The result implied that HCS might benefit patients more than PCS in non-displaced fractures. For the non-displaced fractures with relatively limited geological change, HCS could perform better. Another biomechanics study mentioned that HCS could obtain compression between bone fragments only if the initial gap is less than the gap closed, and the fragment compression might be immediately lost if the screw is reversed^47^. For the displaced fractures, the initial gap between bone fragments was hard to control, once the gap was not closed, the compression would not be possible, and the adjustments would also result in the loss of compression. In our study all reductions were performed minimally invasively, which could possibly leave little gaps between the fragments. Our reduction evaluation was the Garden alignment index which could not reflect the gap between the fragments. This might explain why HCS group had longer union time and higher union rate. Dr Zhang et al. suggested stronger fixation in the unstable vertical fractures due to the better performance of implant in a biomechanics study^42^, but the healing of femoral neck fracture is a special process with a relative long time period, a consistent big stress and a higher risk of shortening and necrosis. Although HCS might protect the femoral neck from varus and shortening in the beginning, it would be difficult for the screw to stay firm in the long run. The stress concentration on the screws could loosen the fixation. On the contrary, PCS screws might avoid the severe complications of screw cut-out and nonunion by complying the geometric change of femoral neck and could conduct the constant compression. Once HCS cannot maintain the original position, the complications would emerge. Thus, the more appropriate use case for HCS might be non-displaced fractures. For the bone mineral density, we were not able to collect the BMD value of every patient. We tried to use the CTI to represent BMD, but no significant relationship was found between the CTI and all prognoses. The reliability of CTI was still controversial, especially for the fracture patients^48^. Hence, we leave the consideration of BMD for future studies.

Second, the cut-out and back-sliding of the screw were not observed in Dr. Ocku’s study. Screw cut-out and back-sliding were important in the comparison between the two groups. The length of the screws remained still when the femoral neck shortening happened. The screws would be over length in either direction. It was understandable that PCS had more back-slidings and less cut-outs. Screw cut-outs could be painless in the early stage but would result in severe consequence in the long run. HCS prevented screw back-sliding in the non-displaced fracture patients but resulted in worse outcomes in the displaced fractures. Without the subgroup analysis, the differences between different groups would be neglected.

Third, the criterion of the angle change and shortening in Dr. Okcu et al.’s study was broader. Although the shortening of 10 mm and angle decrease of 10 degrees were commonly used in other studies, the differences for a research of one year follow-up could be slight. By narrowing the criterion, we could better compare the differences between groups.

Fourth, the limited sample size and unblinded design in their study could result in unknown biases.

^44^. They mainly focused on the complication rates, femoral neck shortening and the change of neck shaft angle. They found that the outcomes were similar between the two groups and drew the conclusion that HCS could not provide a length-stable fixation in non-displaced FNF. However, they did not observe the movement along the axis of the screws, and they did not categorize the continuous variable to reveal the potential differences. Moreover, the function scores were missing, which was the significant result found in our study.

Dr. Zhang et al. confirmed the better biomechanical stability of HCS than PCS especially in the vertical fracture models^42^. They also combined the two different screws as a new configuration of fixation and received good results^43^. This new configuration could be a solution to combine the advantages of the two kinds of screws but need more comparison studies with three HCS and other configuration of screws.

Other studies varied a lot. The fully threaded screws used in these studies were not headless nor with variable pitch. They played assistant roles with other fixation methods. The outcomes in most studies were good but there was a lack of control groups^35,45,46^. The use of HCS in the fixation of FNF was still controversial. Studies on when and how to use these screws are in great need.

In our results, the length stable and angle stable characteristics of HCS were obvious compared with PCS, which was in line with the previous biomechanics literature. Our study extends the previous literature in the following ways. This study was one of the few clinical comparative studies on the use of HCS in the treatment of femoral fracture. Compared to other studies, additional information of influencing factors and prognoses were collected. For example, the prognosis of screw cut-out was considered, which has not yet been studied before. Detailed and convenient imaging measurements were done and significant differences between groups were identified. Furthermore, we found fracture displacement important to the choice of different kinds of cannulated screws, which has not been mentioned in the former studies either. Besides, the proposed X-ray measurement method could provide potential reference for future research, and the multivariate analysis and subgroup analysis could reveal the characteristics of HCS from different point of views. This research contributed to the better understanding of HCS and answered the question on when and how should the screw be used.

However, limitations of this study exist. First, this study was retrospective, and the sample size was relatively small. Many confounding factors may exist. Age might confound with fracture classification, BMD and etc. Due to the retrospective nature of the study, the standard of how patients were allocated into different fixation groups was not unified. For the included cases performed by different surgeons, the first grouping factor should be the surgeon’s subjective preference. Second, HCS was thought to be biomechanically stronger than PCS and can reduce femoral neck shortening, thus the surgeons might prefer to use HCS in high-risk femoral neck shortening patients. However, the evaluation of risk was subjective with BMD and fracture details estimated based solely on X-rays. At last, the included two groups showed no significant difference in the descriptive statistics, which means that the surgeon’s preference has no significant impact on grouping. Therefore, it will not affect the results and interpretations of this study. No significant result was found about age, gender, BMI, CTI, time from injury to operation and length of hospital stay in the univariate, multivariate and stratified analyses. As a result, no conclusion on these factors could be drawn. We plan to conduct a randomized control study in the near future, however, this study was a preliminary one and proved the new treatment to be harmless. Second, the operators consist of several different surgeons. Although they were all qualified and experienced, apart from difference in fixation selection preference, unknown bias could exist. Third, we lacked the BMD values, and the potential relationship between the BMD and the prognoses could not be detected. The performances of screws under different BMD levels were unknow in this study. Fourth, the preoperative CT scans were unavailable in some cases, and the fracture pattern could not be assessed or classified thoroughly. We planned to fulfill the preoperative CT scans in every future case. At last, we followed up for only one year and the long-term prognoses were unknown.

## Conclusions

Compared with PCS, HCS had a lower screw back-sliding rate, femoral shortening rate, angle decrease rate and similar function score, but would result in more screw cut-outs in displaced FNF. As a conclusion, HCS is not recommended to be used in displaced FNF due to its higher screw cut-out rate, and its potential advantage in non-displaced FNF needs to be further proved. Further qualified investigations with a larger scale of patients and longer follow-up are needed in the future.
